# Effects of variable retention harvesting on canopy transpiration in a red pine plantation forest

**DOI:** 10.1186/s13717-022-00366-0

**Published:** 2022-03-18

**Authors:** Alanna V. Bodo, M. Altaf Arain

**Affiliations:** 1 School of Earth, Environment and Society, McMaster University, 1280 Main Street West, Hamilton, ON L8S 4K1 Canada; 2 McMaster Centre for Climate Change, McMaster University, 1280 Main Street West, Hamilton, ON L8S 4K1 Canada

**Keywords:** Variable Retention Harvesting, Forest management, Transpiration, Sap flow, Red pine, Great Lakes region

## Abstract

**Background:**

Variable Retention Harvesting (VRH) is a forest management practice applied to enhance forest growth, improve biodiversity, preserve ecosystem function and provide economic revenue from harvested timber. There are many different forms and compositions in which VRH is applied in forest ecosystems. In this study, the impacts of four different VRH treatments on transpiration were evaluated in an 83-year-old red pine (Pinus *Pinus resinosa*) plantation forest in the Great Lakes region in Canada. These VRH treatments included 55% aggregated crown retention (55A), 55% dispersed crown retention (55D), 33% aggregated crown retention (33A), 33% dispersed crown retention (33D) and unharvested control (CN) plot. These VRH treatments were implemented in 1-ha plots in the winter of 2014, while sap flow measurements were conducted from 2018 to 2020.

**Results:**

Study results showed that tree-level transpiration was highest among trees in the 55D treatment, followed by 33D, 55A, 33A and CN plots. We found that photosynthetically active radiation (PAR) and vapor pressure deficit (VPD) were major controls or drivers of transpiration in all VRH treatments. Our study suggests that dispersed or distributed retention of 55% basal area (55D) is the ideal forest management technique to enhance transpiration and forest growth.

**Conclusions:**

This study will help researchers, forest managers and decision-makers to improve their understanding of water cycling in forest ecosystem and adopt the best forest management regimes to enhance forest growth, health and resiliency to climate change.

## Introduction

Afforestation and reforestation are widely adopted practices to enhance carbon sequestration and mitigate climate change (Pan et al. 2011; Law et al. 2018; Domke et al. 2020). However, forest managers and planners are challenged with appropriately managing these forests. They employ various silvicultural treatments to sustain or enhance forest growth and emulate the ecological functions that are typical of natural forests. In this endeavor, they must balance timber production, carbon sequestration and biodiversity aspects while preserving ecosystem health (Martínez Pastur et al. 2020). Converting abandoned agricultural and marginal lands to plantation forests can also impact hydrological cycles by increasing infiltration and evapotranspiration that may cause higher local rainfall and changes in regional water cycle (Van Dijk and Keenan 2007; Ellison et al. 2017). Future climate change prediction models suggest an increase in temperature and acceleration of hydrologic cycle with extreme precipitation events, hotter and drier summers and longer growing seasons in many parts of the world, including the Great Lakes region in North America (Zhang et al. 2019). Therefore, enhancing forest cover and adopting better forest management practices in this region may mitigate these effects by increasing infiltration, reducing surface runoff and hence ‘flash’ flooding events and enhancing and stabilizing precipitation, which in turn may prevent sustained periods of drought (Van Dijk and Keenan 2007; Ellison et al. 2017).

Variable Retention Harvesting (VRH) practices are employed worldwide to address silvicultural objectives and protect and enhance forest ecosystem services. VRH often aims to increase resilience by applying partial cutting treatments, leaving single or small groups of trees (Bladon et al. 2006; Gustafsson et al. 2012). In Canada, retention forestry first emerged in British Columbia in the early 1990s and was implemented in more eastern provinces, including Ontario, by the late 1990s (Gustafsson et al. 2012). Today, more than 50% of forestland in Ontario is managed using the retention forestry approach (Gustafsson et al. 2012). In the past, the focus of VRH research has been to assess the impact of these harvesting regimes on wood growth and biodiversity. Some studies, however, have examined the effects on micrometeorological variables and key components of the hydrologic cycle, and have shown that residual trees may benefit from reduced resource competition (Wang et al. 1995; Liu et al. 2003; Skubel et al. 2017). Bladon et al. (2006) reported an increase in wind speed, net radiation, soil water content and vapour pressure deficit following VRH, which contributed to higher rates of transpiration in some species. In addition to hydrological components, tree growth (Bebber et al. 2004; Powers et al. 2010; Dwyer et al. 2010) and carbon sequestration (Zugic et al. 2021) have also been shown to increase in residual trees following VRH.

**Fig. 1:**
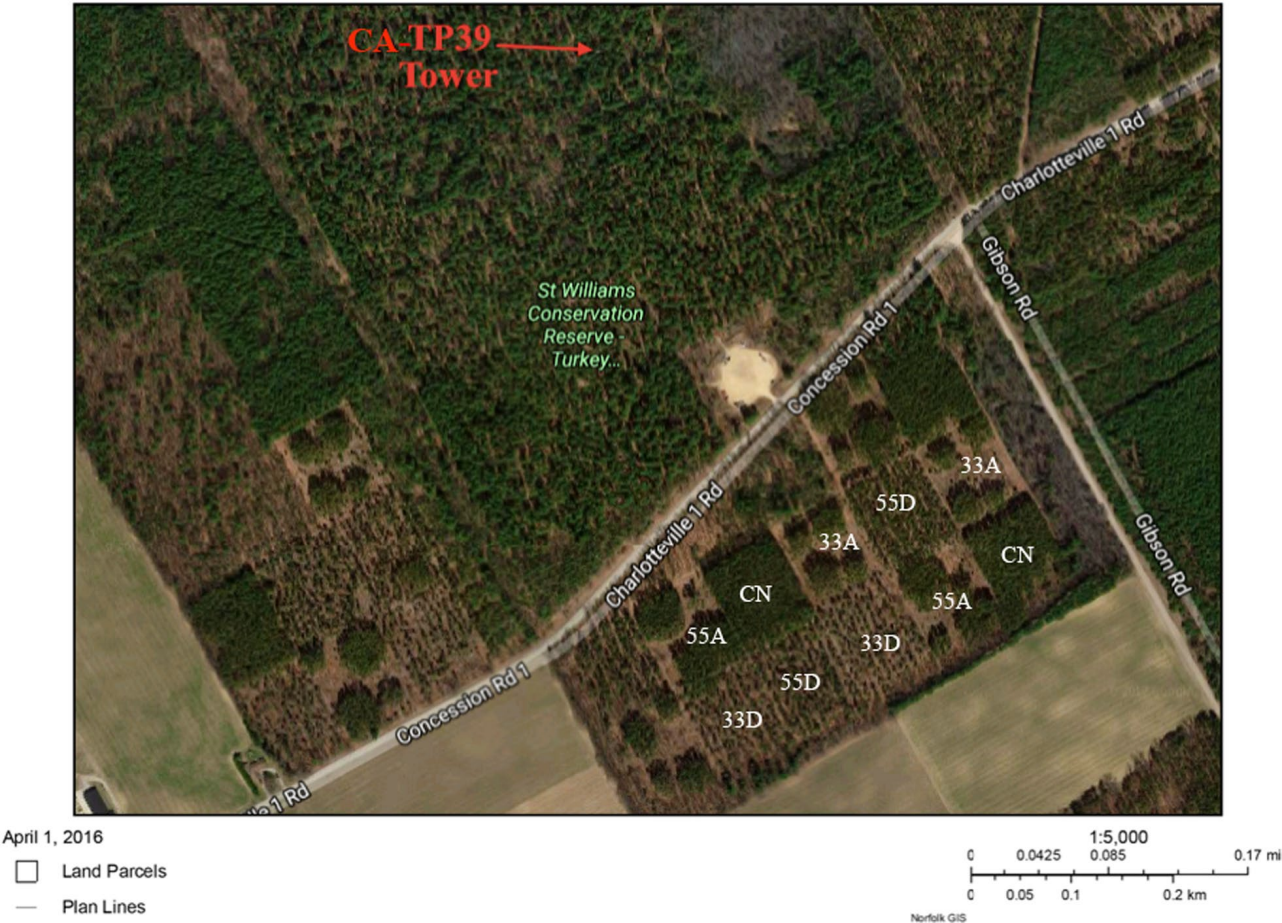
Aerial photograph of CA-TP39 and CA-TP31 study area obtained from Google Earth 2016 showing the various 1-ha VRH treatments

In the Great Lakes region in Southern Ontario, Canada, five different harvesting treatments were applied in a 21-ha 83-year-old red pine plantation stand by the Ontario Ministry of Natural Resources and Forestry (OMNRF) in the winter of 2014 with the ultimate goal of restoring this monoculture pine stand to native mixed forest. These VRH treatments included 55% aggregated crown retention (55A), 55% dispersed crown retention (55D), 33% aggregated crown retention (33A), and 33% dispersed crown retention (33D) and unharvested, control plots (CN). The dispersed method removed trees in an even formation, whereas the aggregated crown retention involves leaving the remaining un-harvested trees in small or large groups (Fig. 1). The 55 and 33% corresponds to the percentage of basal area retained after harvesting. This experimentally managed forest is the first research application of VRH in Ontario, and the first experiment anywhere to restore a red pine plantation to native forest type using VRH.

We hypothesize that sap flux density will be highest among the most heavily thinned (33% retention) treatments, due to a reduced competition for water resources. At the tree-level, we hypothesize that the 33D and 55D treatments will have the highest daily transpiration due to their larger conductive sapwood area and increased growth after thinning. The results of this study will help to develop a better understanding of the effects of silvicultural practices on the overall water balance of plantation forests.

Furthermore, this study is one of the first to examine the effects of VRH on transpiration in red pine (*Pinus resinosa*) ecosystems. An estimated 70% of plantation forests in the Southern Ontario or Great Lakes region are red pine stands, which are an important source of revenue from harvested timber (Kim 2020). Red pine lumber is used for pulp wood and electricity and other utility poles. Therefore, the establishment of these plantation forests not only helps to fulfill timber demand, but also provides a natural solution for the mitigation of climate change, while conserving forest ecosystems and watershed hydrologic functions (LRC 2005).

This study aims to determine the effect of VRH treatments on sap flow and transpiration among remaining dominant canopy trees in each treatment and the control plot. Investigating these differences would help researchers to explore the effects of these thinning treatments on the growth and hydraulic functionality in managed forests and their resilience to climate change. The specific objectives of this study are (i) compare sap flux density or transpiration between five different VRH treatments and (ii) determine the major environmental drivers of transpiration to better understand the response of managed forests to environmental changes.

## Methods

### Experimental site description

This study was conducted in a temperate red pine (*Pinus resinosa*) plantation stand in the St. Williams Conservation Reserve (SWCR) (42°42′N, 80°21′W), located 3.0 km north of Lake Erie, in Southern Ontario, Canada. This 21-ha red pine plantation stand is part of the Turkey Point Observatory (TPO) and is referred to as CA-TP31, as the stand was planted in 1931. Topography at this site is predominantly flat with an elevation of 184 m.TPO sites are also associated with the Global Water Futures Program and global FLUXNET. Further details of the Turkey Point Observatory are provided in Restrepo and Arain (2005), Peichl et al. (2010), Beamesderfer et al. (2020) and Arain et al. (2022).

CA-TP31 was established by planting seedlings 2 m apart, in furrowed rows (~ 2500 trees/ha). This monoculture plantation was subject to thinning in about 1960–1961 that reduced stand density to about 1875 trees/ha (McKenzie et al. 2021). As mentioned earlier, in this stand, a variable retention harvesting (VRH) regime was employed in the winter of 2014 by the Ontario Ministry of Natural Resources and Forestry (OMNRF) to evaluate the effectiveness of five different VRH treatments (33A, 33D, 55A, 55D and Control) on forest growth and ultimately restore this plantation stand to a native forest ecosystem. Each VRH treatment included a 1-ha plot with three replicates where different harvesting densities and patterns were applied, as shown in Fig. 1. The percentage of basal area retained in each VRH treatment plot is given in Table 1.

**Table 1:** Variable Retention Harvesting treatments in TP31

Plot abbreviation	% basal area retained after thinning	Pattern of thinning	Stand Density (trees plot^−1^)	Average DBH (m)	Average As (m^2^)
33A	33	Aggregate	178	0.311	0.0609
33D	33	Dispersed	118	0.296	0.0542
55A	55	Aggregate	213	0.279	0.0472
55D	55	Dispersed	235	0.305	0.0582
CN			432	0.278	0.0468

Local micrometeorological conditions were measured from two flux towers located at the white pine (*Pinus strobus*) plantation sites (CA-TP39, CA-TP74) within a 2-km radius of CA-TP31. These towers are instrumented with closed-path eddy-covariance systems and weather stations, where continuous half-hourly fluxes and meteorological measurements (carbon dioxide, sensible and latent heat fluxes, four components of radiation, photosynthetically active radiation (PAR), air temperature, humidity, windspeed and direction, soil temperature, and soil moisture) have been conducted, since 2003 (Arain et al. 2022). Soil in this region is sandy and well-drained (McLaren et al. 2008; Beamesderfer et al. 2020). The climate in Southern Ontario is temperate with warm, humid summers and very cold winters. Mean annual temperature is 8.0 C and the area receives on average 1036 mm of precipitation each year, approximately 13% of which falls as snow (Environment Canada, 1980–2010 Norms at Delhi, ON).

### Sap flow measurements

Sap flow sensors were installed in five plots, representing each of the four treatment types and the un-thinned control plot. Eight trees within each plot were instrumented with one sap flow sensor in the outermost 20 mm of sapwood. Sample trees were randomly selected based on overall health (e.g., full crown, undamaged bark) and on proximity to the data logger and power supply (less than 30 m away). The sensors were self-manufactured, Granier-style thermal-dissipation (TD) sensors following Matheny et al. (2014) and Pappas et al. (2018). Each sensor consisted of two hollow needles, 20 mm in length, each containing a fine-wire, type T thermocouple at the midpoint (10 mm) of each needle. One of the needles was wrapped with insulated, constantan wire, which provided constant heating when connected to the self-made circuit board and supplied 12 V power. The needles were coated with thermal grease and inserted into a hollow, metal tube on the north side of the tree at breast height (1.3 m above the ground). The heated probe was installed 10 cm vertically above the non-heated probe.

In each sample tree, one sensor was installed in the outer-most 0–20 mm of sapwood, except for 5 trees which were equipped with radial sensors for the measurement of non-uniform flow (Bodo and Arain 2021). Raw measurements (mV) were collected with 30 min resolution on a CR10X datalogger (Campbell Scientific, Logan, UT, USA) continuously from 1 July 2018 to 31 October 2020 and averaged into half-hour intervals. A dimensionless flow index (*K*), was calculated from the difference in temperature (*T*) measured between the two probes following Granier (1987) and can be expressed as

1 $$K=\frac{\Delta \mathrm{Tmax}-\Delta T}{\Delta T}$$

where *T* represents temperature in degrees Celsius. *K* values were calculated by determining zero-flow conditions using the double regression method in R package TREX (Tree sap flow Extractor; R Core Team 2017) developed by Peters et al. (2021). All sampled trees were located within 30 m radius from the datalogger box and power supply due to voltage drop considerations. Therefore, in some instances, we were limited in the selection of trees for sapflow sensors in VRH plots, which could cause some uncertainty when comparing treatment types.

### Wounding/signal dampening correction

It is widely known that the insertion of sapflow sensors into tree stems and subsequent heating of sapflow sensor probes may cause ‘wounding’ of stem tissue. Over time tree resin is also deposited over the probes due to healing from the drilled holes where sensors are installed. Because of these effects and when sensors have been installed for multiple years, many studies have reported a dampening in the raw sensor signal (Steppe et al. 2010; Wullschleger and King 2000; Wiedemann et al. 2016) which may lead to significant underestimations. We corrected for underestimations in *J*_*s*_ resulting from signal dampening between sensor installation (2018) and the 2019 growing season by installing two new sensors into the CN plot. Using a linear regression model, we determined a 69% reduction in *J*_*s*_ values in original sensors when compared to newly installed sensors (average *R*^2^ = 0.94). Both the 2019 and 2020 data were corrected to reflect this relationship between time of installation and signal dampening. A correction factor was not determined empirically for 2020 due to field restrictions during the COVID-19 pandemic, so the correction factor was assumed to be the same for both 2019 and 2020.

### Scaling sap flow measurements

Sap flux density (*J*_*s*_; g H_2_O m^−2^ s^−1^) was calculated following Granier (1987). Whole-tree water use (TWU) was calculated following:

2 $$\mathrm{TWU}=J{\text{s}} \, \times A{\text{s}} \times \rho {\text{w}}$$

where *J*_*s*_ is sap flux density (g H_2_O m^−2^ s^−1)^, *A*_*s*_ is the sapwood area of the tree (m^2^) and *ρ*_*w*_ is the density of water (1000 kg m^−3^). TWU was also corrected for radial differences in sap flux density according to Bodo and Arain (2021), which assumes that *J*_s_ is highest in the outermost 20 mm of sapwood and decreases toward the sapwood-heartwood boundary.

### Gap filling and statistical analysis

Due to an unpredicted power failure, data gaps occurred in the control (CN) and 55D plots from 30 August to 22 October 2020. Other minor (a few hours or a few days) gaps also occurred throughout the study period due to sensor failure and power interruptions. Minor gaps in *J*_*s*_ were filled using a linear relationship with environmental variables PAR and VPD. The average correlation coefficient was 0.68 and the minimum coefficient was 0.56 (*p* < 0.01). Gap-filled data were not used to examine meteorological drivers of sap flux density in our analysis.

A linear regression model was used to test the significance of environmental conditions (VPD, Ta, PAR) on sap flux density. The fit of the model and the significance of the regression coefficients were assessed using the *F*-statistic. To test for significant differences in sap flux density among the VRH treatments, an analysis of variance test (one-way ANOVA) was conducted. All statistical analyses were conducted using MATLAB (The MathWorks Inc.).

## Results

### Meteorological conditions

**Fig. 2:**
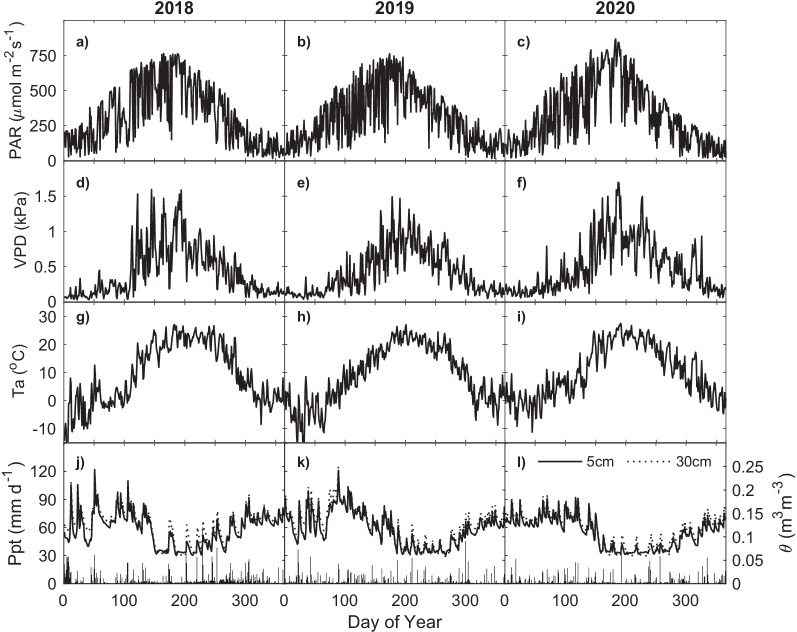
Annual daily average measurements of photosynthetically active radiation (PAR; **a**–**c**), vapour pressure deficit (VPD; **d**, **f**), air temperature (Ta; **g**–**i**); daily total precipitation (Ppt; **j**–**l**) and daily average soil moisture (θ, **j**–**l**) for years 2018, 2019 and 2020

The site received a total of 1644, 1126 and 1056 mm of precipitation in 2018, 2019 and 2020, respectively (Fig. 2j–l). With regard to growing season precipitation only (1 April to 31 October), 2018 was significantly more wet (985 mm) compared to 2019 (677 mm) and 2020 (619 mm). Air temperature throughout the study period was seasonally consistent between years, with the exception of April 2018, where the average monthly temperature was 3.6 °C, compared with April 2019 (6.7 °C) and April 2020 (5.3 °C) (Fig. 2g–i). Additionally, May 2018 (17.1 °C) was strikingly warmer than May 2019 (13.0 °C) and May 2020 (12.7 °C). Finally, we see more prolonged periods of low soil moisture (< 0.1 m^3 ^m^−3^) in 2020 compared to other years (Fig. 2j–l). This is likely due to a combination of factors including higher average temperatures and a lower precipitation in June (19.7 °C) and July (24.0 °C) months on 2020 with a combined total precipitation of 131 mm.

### Effects of VRH on sap flux density

**Fig. 3:**
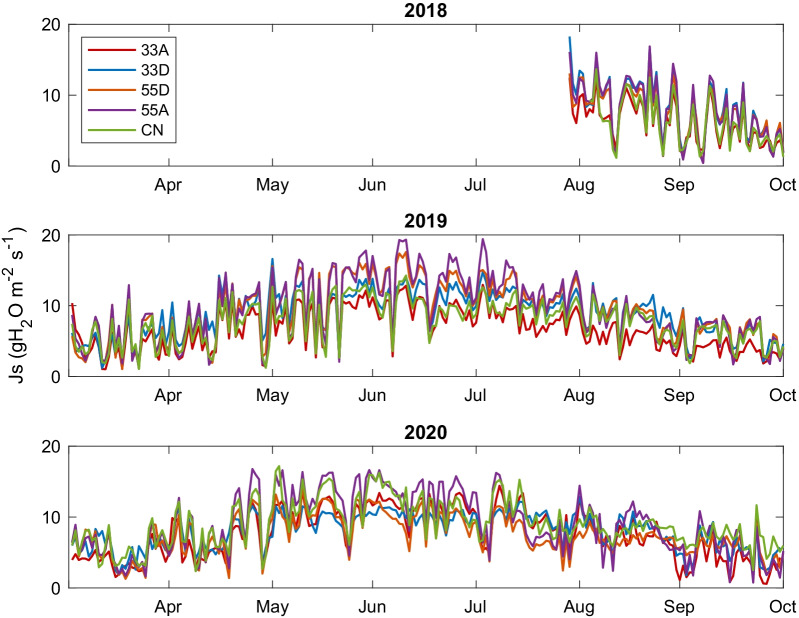
Daily average sap flux density for all plot types for the growing season (1 April to 31 October) for all years

To best describe the results of our study, we divided the growing seasons into three periods, where 1 April to 30 May is referred to as the early growing season, 1 June to 31 August as the mid growing season and 1 September to 31 October as the late growing season. Our results (Fig. 3) revealed small differences in *J*_*s*_ among VRH plots in the late growing season of 2018 and 2019, with the 33D and 55A plots exhibiting the highest *J*_*s*_. However, in the mid growing season (May to July) in 2019, we observed major differences between treatments, with the 55A and 55D plots dominating in terms of *J*_*s*_*.* That year, we saw similar values among all treatment types in the early growing season as well, but differences among the treatments arose in mid-to-late June where we saw that the general trend in daily average *J*_*s*_ was 55A > 55D > 33D > CN > 33A. A similar trend was observed in 2020 where daily average *J*_*s*_ was highest in the 55A, particularly in the early and mid-growing season. With some exceptions, however, in the mid 2020 growing season, we observed higher *J*_*s*_ among trees in the CN plot compared to the 33A, 33D and 55D. Statistically, sap flux density was significantly different between the VRH treatments for all 3 years: 2018 (*p* < 0.01), 2019 (*p* < 0.001), 2020 (*p* < 0.001).

### Differences in tree-level transpiration

**Fig. 4:**
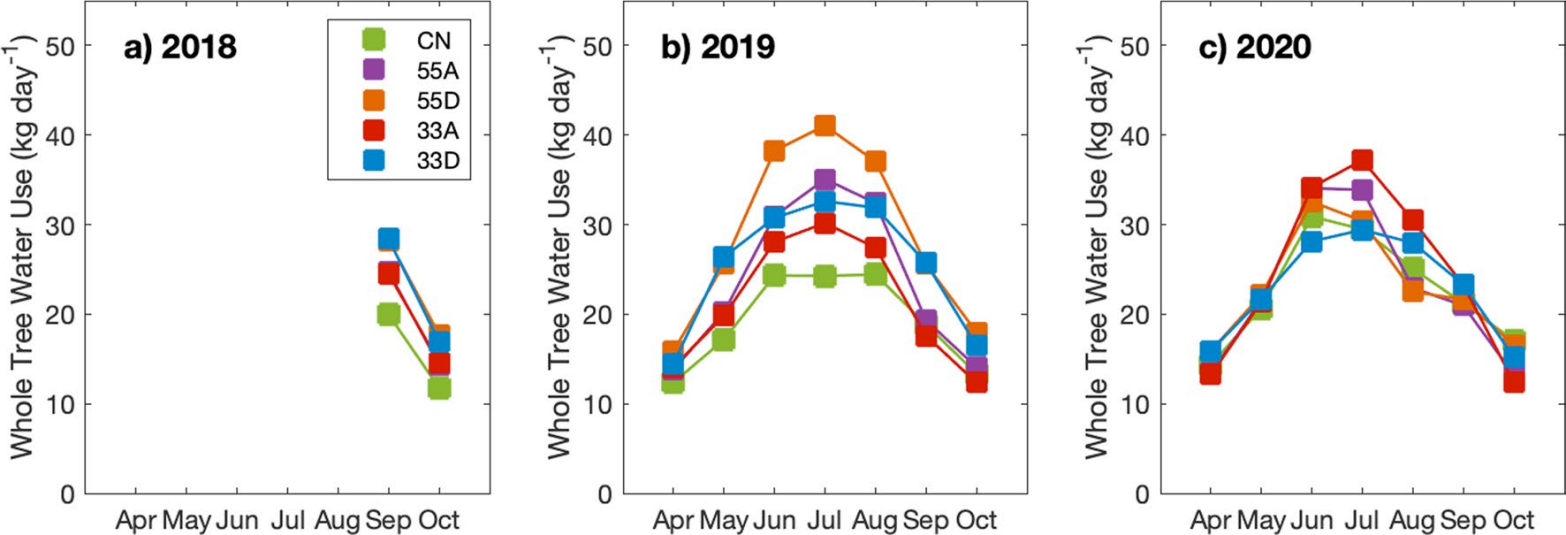
Average daily whole-tree water use by month for 2018 (**a**), 2019 (**b**) and 2020 (**c**)

*J*_*s*_ reflects water flow per unit of sapwood area, which functions well as a method of comparison between treatment types. However, to more accurately compare the water fluxes at the whole tree-level, we quantified tree water-use (TWU) in each VRH treatment for all years of the study (Fig. 4). The results showed a similar general trend between years, with the 55D and 33D treatments exhibiting the highest water-use in 2018, while 55D, 33D and 55A were the highest in 2019 and for most of 2020. Overall, we observed the following trend in TWU: 55D > 33D > 55A > 33A > CN.

### Controls on sap flux density and transpiration

**Fig. 5:**
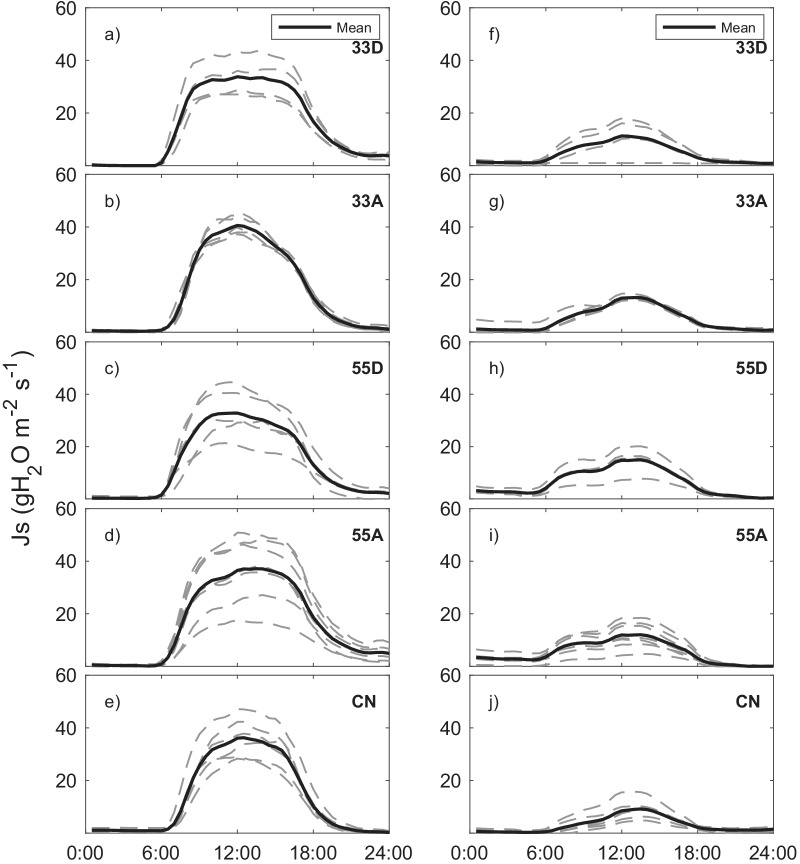
Sap flux density for a 24 h period on a warm, sunny day (5 September 2018; **a**–**e**) and on a cool, cloudy day (12 October 2018; **f**–**j**). Data from individual sensors (6–8 sensors per plot) is shown in the dashed lines and the mean is shown in solid black

Our results showed that both VPD and PAR were the main drivers of *J*_*s*_ among all VRH treatments. We compared diurnal *J*_*s*_ curves for a warm, sunny day (5 September 2018; Fig. 5a–e) and a cool, cloudy day (12 October 2018; Fig. 5f–j). The daily average Ta for these days was 26.7 and 8.2 °C, respectively; and the daily average incoming PAR was 558 and 153 μmol m^−2^ s^−1^, respectively.

**Fig. 6:**
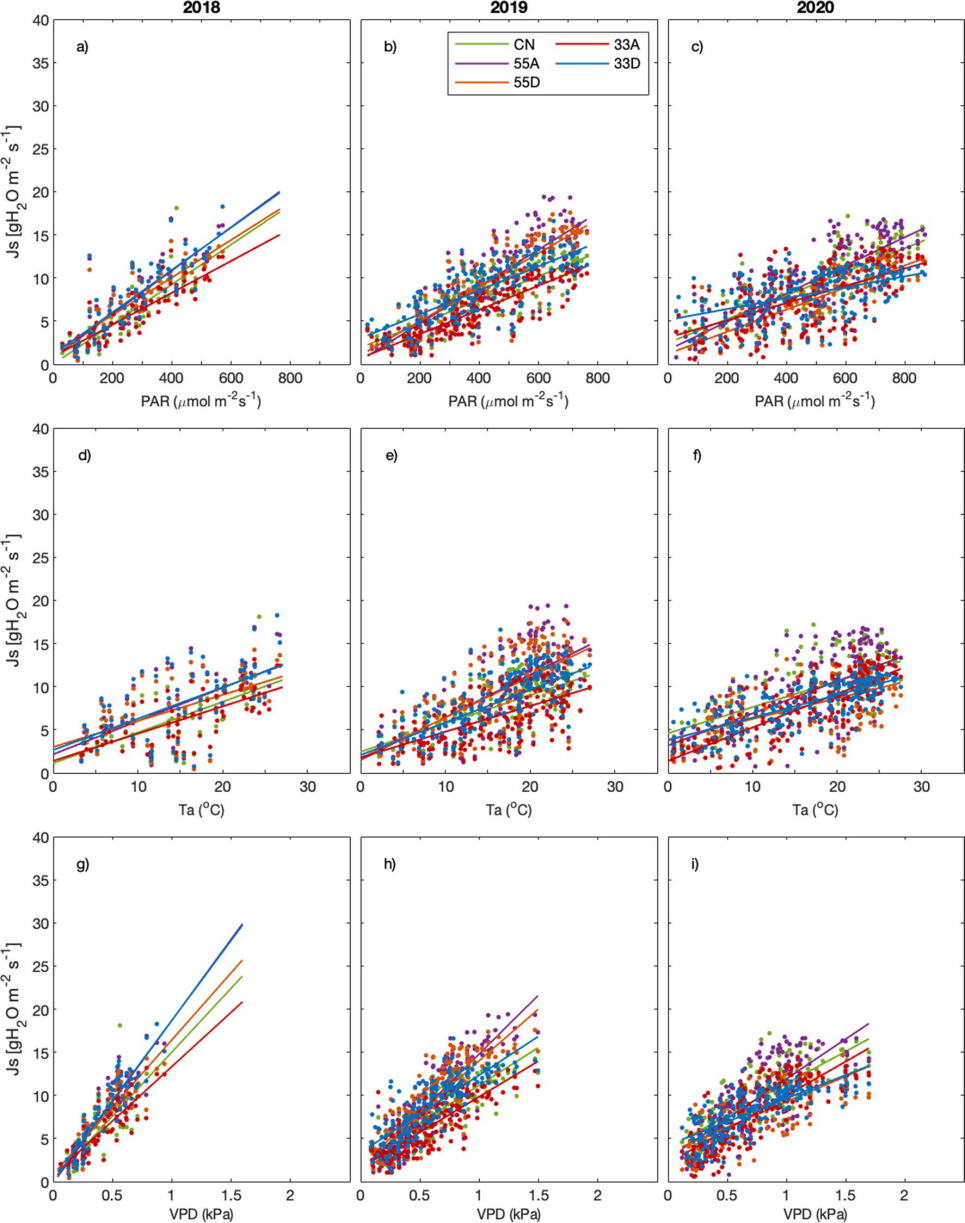
Scatter plots showing the correlation between daily average sap flux density (*J*_*s*_) and photosynthetically active radiation (PAR; **a**–**c**), air temperature (Ta; **d**–**f**) and vapour pressure deficit (VPD; **g**–**i**) for 2018, 2019 and 2020. *R*^2^ values are displayed in Table 2 (*p* < 0.001)

We found that *J*_*s*_ and, by proxy, transpiration was mainly driven by VPD, with some exceptions (Fig. 6). In 2020, during periods of low precipitation, *J*_*s*_ in the 33A and 33D plots were strongly driven by VPD (*R*^2^ values reported in Table 2). By contrast, in 2018, trees in the control and 33A plots were more closely coupled with PAR.

**Table 2:** Correlation coefficients (*R*^2^ values) describing the linear relationship between daily average sap flux density and daily average PAR, Ta and VPD (*p* < 0.001)

	2018			2019			2020		
	PAR	Ta	VPD	PAR	Ta	VPD	PAR	Ta	VPD
33A	0.77	0.51	0.72	0.73	0.37	0.66	0.34	0.65	0.65
33D	0.72	0.34	0.83	0.60	0.50	0.66	0.25	0.54	0.55
55A	0.74	0.39	0.83	0.71	0.42	0.74	0.61	0.40	0.60
55D	0.75	0.33	0.77	0.73	0.47	0.71	0.62	0.35	0.50
CN	0.77	0.44	0.64	0.65	0.41	0.69	0.53	0.43	0.61

## Discussion

It is widely discussed in the literature that a healthy and productive forest provides numerous benefits, such as lumber, carbon sequestration and long-term storage, enhanced biodiversity and better regulation of the hydrologic cycle (Bonan 2008). Red pine forests are a preferred plantation species in the Great Lakes region in Canada and the USA (Kim 2020). Several studies have been conducted in red pine forests in the Great Lakes region to examine their growth, yield and wood productivity in response to thinning treatments or climatic stresses (Bradford and Palik 2009; Magruder et al. 2013). However, none of these studies have examined effects on transpiration. To our knowledge, this study is the first effort to explore the impact of VRH on transpiration in the Great Lakes region. Some scientists have argued that the hydrologic benefits of forests should be considered among the primary contributions of forest ecosystems to mitigating climate change, highlighting the development for adaptation and management policies (Ellison et al. 2017).

### Effects of VRH on sap flux density

Our study showed that thinning reduces water-resource competition among remaining trees, leading to higher tree-level transpiration in the remaining trees. Several understory species have emerged since thinning, and the amount of understory vegetation is positively correlated with the thinning intensity. While we did not measure sapflow in any of the understory species, we acknowledge these plants compete with the dominant red pine for soil water. The significant amount of understory vegetation in the 33A plot may be the cause of lower *J*_*s*_ values in this treatment. Our results from 33A refute the hypothesis that *J*_*s*_ would be highest among trees in the more heavily thinned treatments (e.g., 33A and 33D). It shows the complexities of these linkages and the need for more long-term observations and research. By contrast, we observed higher TWU in 55D and 33D plots, which suggests that a dispersed thinning pattern is favorable to that of an aggregated pattern when concerned with promoting transpiration. Because TWU was quantified by applying *Js* to the total sapwood area, these general patterns more closely follow the trend in average tree sapwood area among the treatments (Table 1). TWU is an important metric for describing water quantities at the tree-level, because although trees in the 55A plot may exhibit the highest *J*_*s*_, when scaled to the tree-level, we observe that 55D and 33D to have the highest TWU or tree-level transpiration.

In another study at our site, using tree ring measurements, Zugic et al. (2021) have found that dispersed VRH treatments at both levels of retention (55D and 33D) were more effective in promoting post-harvest tree-level growth and carbon sequestration than aggregate treatments. However, their study also suggested that at the stand-level after accounting for retained tree biomass, the growth and carbon sequestration were highest for greater levels of retention, regardless of the pattern of treatment. These results support our findings that moderate levels of thinning in a dispersed pattern may promote higher tree-level water-use and hence higher growth, which may help to offset the impacts of a changing climate.

In addition, several other studies have used sapflow sensors to investigate the effect of thinning on sap flux density. For example, Skubel et al. (2017) used sapflow sensors to examine tree- and stand-level transpiration at CA-TP74 site (part of the TPO) following a 13% reduction in basal area (87% retention) from thinning. Their study found an increase in tree-level transpiration among the remaining trees following the moderate thinning event. In a long-term study on Chinese pine (*Pinus tabuliformis*), Chen et al. (2020) reported greater DBH, greater sapwood area and higher transpiration among heavily thinned stands (80% and 65% reduction) when compared to a moderately thinned stand (55% reduction) 30 years after the thinning. By contrast, Park et al. (2018) observed transpiration in Korean pine (*Pinus koraiensis)* following heavy and moderate thinning and reported an increase in transpiration following thinning in both stands; however, the effects of the light thinning decreased over time. The expansive literature on sapflow studies suggests a positive relationship between thinning and tree-level transpiration, but there seems to be no conclusive recommendation for the optimal thinning intensity and pattern and the controls on these processes may be dependent on the tree species and climate of the region. Therefore, our study will be a valuable addition to the sapflow literature.

### Controls on sap flux density and transpiration

Impacts of forest management treatments on transpiration and water cycle are quite complex where various factors may exert opposing controls. In our study, we found that during periods of low precipitation, VPD was the main driver or control on *J*_*s*_ in our VRH treatments (e.g., 33A and 33D). However, *J*_*s*_ was more closely coupled with PAR in the unharvested or control plot (CN). Studies in the literature have shown that thinning increases penetration of radiation in the canopy, while remaining trees in a treatment regime have been shown to be exposed to higher fluctuations in wind speed, air temperature and evaporative demand (Man and Lieffers 1999; Proe et al. 2001; Bladon et al. 2007). Some studies have also shown that thinning may decrease absorption of PAR by the canopy due to the removal of low-albedo coniferous crown cover; however, below canopy transmission of PAR may increase causing higher exposure of understory and ground surface to radiation resulting in higher soil temperatures (Anderson et al. 2010; Cherubini et al. 2018). Although VPD was not measured at the canopy level in each treatment plot, we expect VPD to be lower in the control, due to the closed canopy of this homogeneous plot. These results suggest that the application of VRH in a plantation forest changes the dominant environmental drivers of water flux, where VPD becomes increasingly significant in addition to PAR. Furthermore, we expect the extent of this change to increase with increased levels of thinning which causes a more open canopy and heating of the canopy and ground.

Thinning may also increase soil moisture due to reduced competition and hence higher transpiration in remaining trees (Breda et al 1995; Reid et al. 2006). While soil moisture was not directly measured in each of the VRH plots, it is possible that the closed canopy of the CN plot allowed for greater retention of soil moisture throughout the growing season, particularly in periods with low precipitation. On the other hand, removal of canopy cover decreases interception, potentially allowing for more precipitation to reach the soil surface, which may result in greater soil moisture availability and hence more transpiration. In fact, Kurpius et al. (2003) and Simonin et al. (2007) showed that thinning led to increased throughfall, greater water availability and more energy at the soil surface resulting in greater soil evaporation. Future studies should measure soil moisture in each VRH plot to support exploration of the effects of thinning on water balances in each treatment. In our previous research in a similar-age white pine (*Pinus strobus*) stands at our CA-TP39 site, we have also highlighted the complex nature of soil–vegetation–atmosphere interactions in thinned stands (Skubel et al. 2017). Skubel et al. measured sapflow and soil moisture before and after thinning at the CA-TP39 site which was 74-year-old at the time of measurement and found that soil moisture increased immediately following thinning. Ma et al. (2010) also found that soil moisture and VPD increased after thinning, in treatments of various thinning intensities (14–66% basal area removed). These findings suggest a net positive effect of thinning on soil moisture status in harvested stands. However, some studies have also suggested that more intense thinning may lead to a net decrease in soil moisture in thinned stands (Simonin et al. 2007) due to increased surface radiation and subsequent higher evaporation from soil. In our study, two VRH treatments (33A and 33D) involved the removal of 67% of basal area and we found low *J*_*s*_ or transpiration among the 33A treatment. However, we observed higher tree-level transpiration in the 55D and 33D plot, which suggests that the thinning pattern (dispersed vs. aggregated) has a larger effect on transpiration than thinning intensity alone. In addition, our results support the findings that thinning decreases competition among remaining trees for water resources and increases soil moisture (Ma et al. 2010).

## Conclusion

Appropriate management of coniferous plantations can provide a balance between ecological restoration, economic benefit and regional climate change mitigation. Our study results support the use of VRH as an efficient silvicultural treatment to restore red pine plantations to native forest types, with the added benefit of promoting atmospheric moisture through canopy transpiration. Our results show that tree-level transpiration was highest among trees in the 55D treatment, followed by 33D, 55A, 33A and CN plots, suggesting moderate thinning in a dispersed pattern may be optimal to promote growth and transpiration among remaining trees following VRH. Managed forests will play an increasingly important role in climate change mitigation at regional and global scales. Our study further provides empirical data to support decision-making in the region and highlight the complex nature of soil–vegetation–atmosphere interactions in forest ecosystems.

## Data Availability

The data sets used during this study are available from the authors upon request.

## Abbreviations

33A: 33% Basal retention in aggregated pattern
33D: 33% Basal retention in dispersed pattern
55A: 55% Basal retention in aggregated pattern
55D: 55% Basal retention in dispersed pattern
A_s_: Sapwood area [m^2^]
CA-TP31: Turkey Point red pine plantation forest planted in 1931
CA-TP39: Turkey Point pine plantation forest planted in 1939
CA-TP74: Turkey Point pine plantation forest planted in 1974
CA-TP02: Turkey Point pine plantation forest planted in 2002
CA-TPD: Turkey Point mixed deciduous forest stand
CN: Control treatment
DBH: Diameter at Breast Height [m]
Js: Sap flux density [mL m^−^^2^ s^−^^1^]
Ha: Hectare
K: A dimensionless flow index describing the relationship between average flow and zero flow (nighttime) conditions
OMNRF: Ontario Ministry of Natural Resources and Forestry
PAR: Photosynthetically Active Radiation [µmol m^−^^2^ s^−^^1^]
SWCR: St. Williams Conservation Reserve
T: Temperature [°C]
TD: Thermal Dissipation
TWU: Tree Water Use [kg day^−^^1^ or L day^−^^1^]
VPD: Vapour Pressure Deficit [kPa]
VRH: Variable Retention Harvesting
